# Anti-SARS-CoV-2 Activity of *Ampelozizyphus amazonicus* (Saracura-Mirá): Focus on the Modulation of the Spike-ACE2 Interaction by Chemically Characterized Bark Extracts by LC-DAD-APCI-MS/MS

**DOI:** 10.3390/molecules28073159

**Published:** 2023-04-01

**Authors:** Mariana Freire Campos, Simony Carvalho Mendonça, Evelyn Maribel Condori Peñaloza, Beatriz A. C. de Oliveira, Alice S. Rosa, Gilda Guimarães Leitão, Amanda R. Tucci, Vivian Neuza S. Ferreira, Thamara Kelcya F. Oliveira, Milene Dias Miranda, Diego Allonso, Suzana Guimarães Leitão

**Affiliations:** 1 Programa de Pós-Graduação em Biotecnologia Vegetal e Bioprocessos, Centro de Ciências da Saúde, Universidade Federal do Rio de Janeiro, Rio de Janeiro 21.941-902, RJ, Brazil; camposmariana@biof.ufrj.br; 2 Departamento de Produtos Naturais e Alimentos, Faculdade de Farmácia, Universidade Federal do Rio de Janeiro, Ilha do Fundão, Centro de Ciências da Saúde, Rio de Janeiro 21.941-902, RJ, Brazil; sy2802@ufrj.br (S.C.M.); evepharma@ufrj.br (E.M.C.P.); beatrizalbuquerque@ufrj.br (B.A.C.d.O.); 3 Laboratório de Morfologia e Morfogênese Viral, Instituto Oswaldo Cruz, Fiocruz, Rio de Janeiro 21.941-902, RJ, Brazil; alicerosa@aluno.fiocruz.br (A.S.R.); amanda.tucci@ioc.fiocruz.br (A.R.T.); vnsantos@bioqmed.ufrj.br (V.N.S.F.); mmiranda@ioc.fiocruz.br (M.D.M.); 4 Programa de Pós-Graduação em Biologia Celular e Molecular, IOC-Fiocruz, Rio de Janeiro 21.941-902, RJ, Brazil; 5 Instituto de Pesquisas de Produtos Naturais, Universidade Federal do Rio de Janeiro, Rio de Janeiro 21.941-902, RJ, Brazil; ggleitao@ippn.ufrj.br; 6 Departamento de Biotecnologia Farmacêutica, Faculdade de Farmácia, Universidade Federal do Rio de Janeiro, Rio de Janeiro 21.941-902, RJ, Brazil

**Keywords:** Rhamnaceae, saponins, chalcone glycosides, flavonoids, COVID-19, spike protein, coronavirus, mass spectrometry

## Abstract

Traditional medicine shows several treatment protocols for COVID-19 based on natural products, revealing its potential as a possible source of anti-SARS-CoV-2 agents. *Ampelozizyphus amazonicus* is popularly used in the Brazilian Amazon as a fortifier and tonic, and recently, it has been reported to relieve COVID-19 symptoms. This work aimed to investigate the antiviral potential of *A. amazonicus*, focusing on the inhibition of spike and ACE2 receptor interaction, a key step in successful infection. Although saponins are the major compounds of this plant and often reported as its active principles, a polyphenol-rich extract was the best inhibitor of the spike and ACE2 interaction. Chemical characterization of *A. amazonicus* bark extracts by LC-DAD-APCI-MS/MS before and after clean-up steps for polyphenol removal showed that the latter play an essential role in maintaining this activity. The effects of the extracts on viral replication were also assessed, and all samples (aqueous and ethanol extracts) demonstrated in vitro activity, inhibiting viral titers in the supernatant of Calu-3 cells after 24 hpi. By acting both in the SARS-CoV-2 cell entry process and its replication, *A. amazonicus* bark extracts stand out as a multitarget agent, highlighting the species as a promising candidate in the development of anti-SARS-CoV-2 drugs.

## 1. Introduction

Three years have passed since COVID-19 was declared a pandemic. Currently, confirmed cases have reached more than 600 million and led to six million deaths worldwide [1]. SARS-CoV-2, the etiologic agent of COVID-19, is a single-stranded positive-sense RNA virus with a 27–32 kb-long genome [2,3], which is divided into two open reading frames (ORFs) that encode non-structural proteins and subgenomic RNAs that encode the structural proteins [4,5]. Like other enveloped viruses, coronaviruses have unique proteins responsible for the recognition of and binding to host cell receptors [6,7]. The spike protein (SP) has 180 to 200 kDa and is a structural glycoprotein present in all coronaviruses and which is formed by a signal peptide and two subunits: S1, which contains the receptor binding domain (RBD), and S2, in which the fusion peptide is present [8,9]. They are found in the homotrimeric form, as a crown-like ornamentation around the viral particle, which gave the name to this group of viruses [9].

In the viral particle, SP exists in its pre-fusional conformation, and its specific cleavage is mandatory to trigger the conformational change that leads to viral and cellular membrane fusion [9,10]. Two possible entry pathways into host cells have already been described, and both start with the recognition and binding of SP RBD to its major ligand, the angiotensin-converting enzyme 2 (ACE2) [9,10].

Although the vaccine arsenal is widely available to the population and complete vaccination clearly reduces severe cases, it is still imperative to consider mild and moderate infections, whose needs are currently unmet. Controlling non-severe cases will help to reduce the viral spread and reduce the social and economic impact of COVID-19. Therefore, investments in new therapeutic approaches are welcome.

Since the pandemic’s beginning, natural products’ relevance as a potential source of active compounds in the fight against SARS-CoV-2 has exponentially grown, and records in the literature show how these substances represent a promising approach. Plants’ secondary metabolites, such as saponins, terpenes, alkaloids, and phenolic acids, have been shown to display antiviral activity against several specimens of the *Coronaviridae* family [11,12,13]. To date, a few works have described several representatives from different classes of natural products, such as flavonoids and alkaloids, that clearly exhibit a promising activity against SARS-CoV-2 [14,15,16,17], reinforcing the necessity of continued efforts to find potential alternatives to be used in the clinical management of mild and moderate COVID-19 cases.

In 2020, a great number of in silico studies emerged as an alternative to evaluate natural products’ ability to act as protease, helicase, and replicase inhibitors, especially because of the limited infrastructure to manipulate SARS-CoV-2. In silico analyses provided valuable and reliable information on a range of substances with potential anti-SARS-CoV-2 activity in a relatively short time; these are currently being tested in in vitro and in vivo assays [18,19,20,21,22]. Despite viral enzymatic inhibition being the main mechanism of action for most of the currently available and in development antivirals, blockage of SP interaction with ACE2 is becoming an attractive approach for a new generation of antivirals since it has the potential to hamper viral recognition and infection of host cells, preventing viral propagation inside the organism as well as contributing to a reduced mutation rate and thus the appearance of new variants [23].

Lately, in vitro methodologies using isolated viral components have gained space in an attempt to increase the rate of the discovery of potential inhibitors, whether they come from natural or synthetic sources. Some of the technologies used for this purpose include pseudoviruses to investigate the cell entry process, FRET (fluorescence resonance energy transfer) assays to evaluate enzymatic activity, and colorimetric or luminescent assays to evaluate protein interactions [24].

As part of a broader ongoing screening project on natural inhibitors of the interaction between SP and ACE2, a library of plant extracts belonging to Brazilian biome biodiversity (in the Amazon, Atlantic Forest, and Cerrado region) was evaluated, from which the extracts from *A. amazonicus* Ducke stood out as the most promising. *A. amazonicus* is one of 984 species of the Rhamnaceae Juss. family [25], which is endemic to the Amazonian states of South America; its distribution includes the regions of Peru, Colombia, Venezuela, Guyana, Suriname, French Guiana, and Brazil [26,27,28]. The bark and roots of the plant, popularly known as “cerveja de indio” or “saracura-mirá”, have been used in Brazil for over 30 years by the Amazonian traditional communities to prepare a drink with stimulant, tonic and fortifying properties to combat and prevent malaria, among other uses [29]. Studies with a spray-dried aqueous bark extract of the plant have demonstrated its properties as an immune enhancer and adaptogen [30,31]. More recently, the species has been recorded as being used for the prevention and treatment of COVID-19 by the Sateré Mawé indigenous people, as well as by riverine communities in the Brazilian Amazon during the pandemic [32]. Reports on the chemical composition of the plant focus basically on its triterpenes and saponins, which are believed to be responsible for the wide range of biological activities reported for the species [29,30,31,33,34,35]. In fact, aqueous extracts prepared from the plant produce abundant foaming, due to the presence of up to 48% of saponins [31,33], which explains one of the popular names “cerveja de indio” which means “Indian beer”. However, although never studied, phenolic compounds are also present in these plant extracts, especially ethanol compounds (personal observations).

Since the literature shows the potential of *A. amazonicus* as a source of useful compounds for the attenuation of COVID-19 symptoms, we aimed in the present study to evaluate the potential of its chemically characterized bark extracts to inhibit the receptor-binding domain (RBD) of SARS-CoV-2 spike protein and ACE2 interaction before and after clean-up steps with lead acetate for the removal of phenolics, and to inhibit SARS-CoV-2 replication in vitro.

## 2. Results and Discussion

### 2.1. A. amazonicus Extracts Inhibit at Least Half of RBD:ACE2 Complex Formation In Vitro

**Figure 1:**
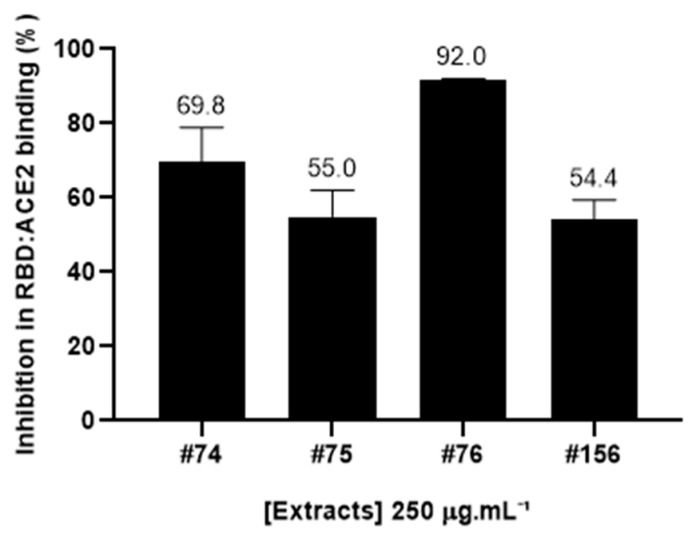
Inhibition rates (in percentage) of the RBD:ACE2 complex of *A. amazonicus.* Aqueous and ethanol bark extracts (250 μg·mL^−1^) were assessed with the Lumit™ immunoassay (Promega). #74—Aqueous bark extract, prepared on a laboratory scale to mimic the traditional beverage; #75—Ethanol bark extract, obtained from the remaining “cake” after the aqueous extraction of the plant material which generated extract #74. #76—Ethanol bark extract, prepared by percolation; #156—Aqueous bark extract, prepared in a pilot-scale industrial plant, according to the traditional method. These data are the result of three independent technical replicates.

The ability of four different extracts of *A. amazonicus* to interfere in the formation of RDB:ACE2 complex was assessed with the aid of a Lumit™ kit (Promega), a no-wash, add-and-read bioluminescence-based kit suitable for the trial of potential inhibitory samples [36].The tested samples included two aqueous extracts prepared according to traditional medicine, which were previously shown to have immunomodulatory and anti-inflammatory properties [30,31], and two ethanol extracts obtained according to the literature [34,35]. At 250 μg·mL^−1^, all the available extracts were able to inhibit at least 50% of the interaction between RBD and ACE2 (Figure 1).

The most active extract (#76) was able to inhibit 92 ± 0.03% of this interaction at this concentration. Notably, a very similar inhibition pattern can be observed for both aqueous extracts (#74 and #156) and ethanol extract #75, which was prepared from the resulting cake after the aqueous extraction of the barks, while ethanol extract #76 was obtained from direct extraction of the plant material with ethanol [34].

### 2.2. Chemical Characterization of A. amazonicus Bark Extracts Reveal the Presence of Saponins, Triterpenes, and Phenolic Compounds

**Figure 2:**
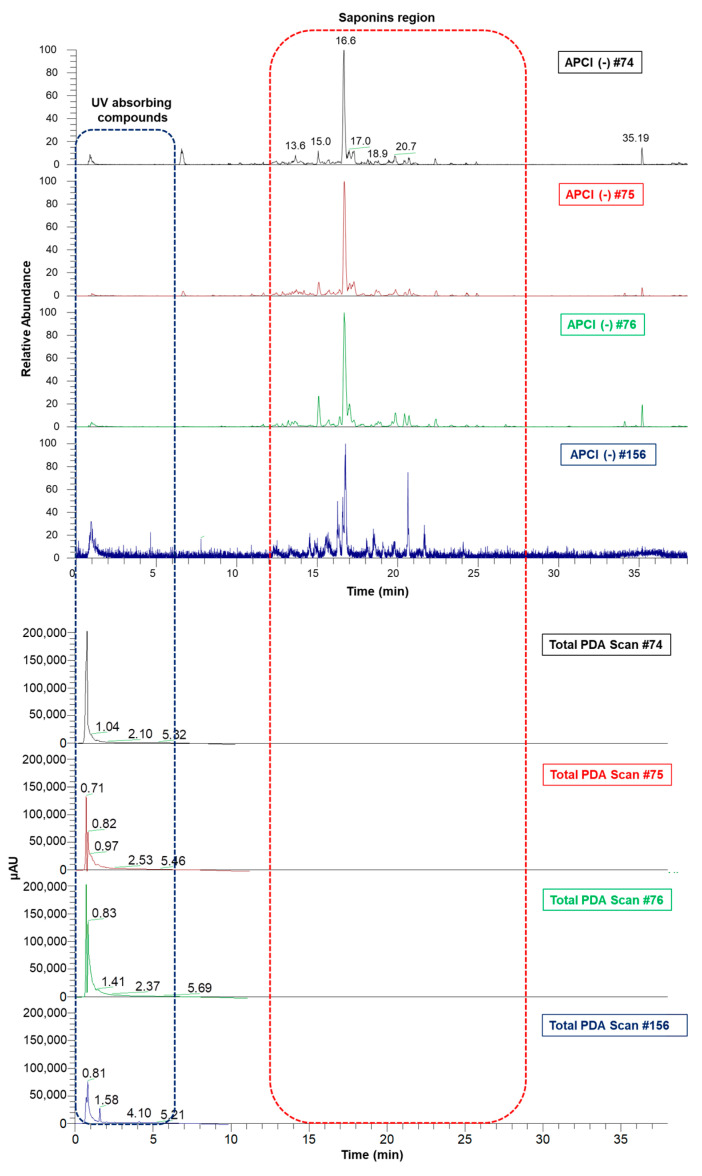
Aligned chromatograms of aqueous and ethanol extracts obtained by UHPLC-PDA-MS/MS. #74—Aqueous bark extract prepared on a laboratory scale; #75—Ethanol bark extract, obtained from the remaining “cake” after the aqueous extraction of the plant material which generated extract #74; #76—Ethanol bark extract, prepared by percolation; #156—Aqueous bark extract, prepared in a pilot-scale industrial plant.

The aqueous (#74 and #156) and ethanol extracts (#75 and #76) of *A. amazonicus* were analyzed by ultra-high-performance liquid chromatography with PDA and coupled to mass spectrometry (UHPLC-PDA-APCI-MS/MS). All extracts showed a very similar saponin profile (Figure 2), which is the main class of compounds described for the species, as reported before [31,34,35,37,38], but a different profile of UV absorbing compounds (as recorded by the PDA detector) not yet reported in the literature for this species.

**Figure 3:**
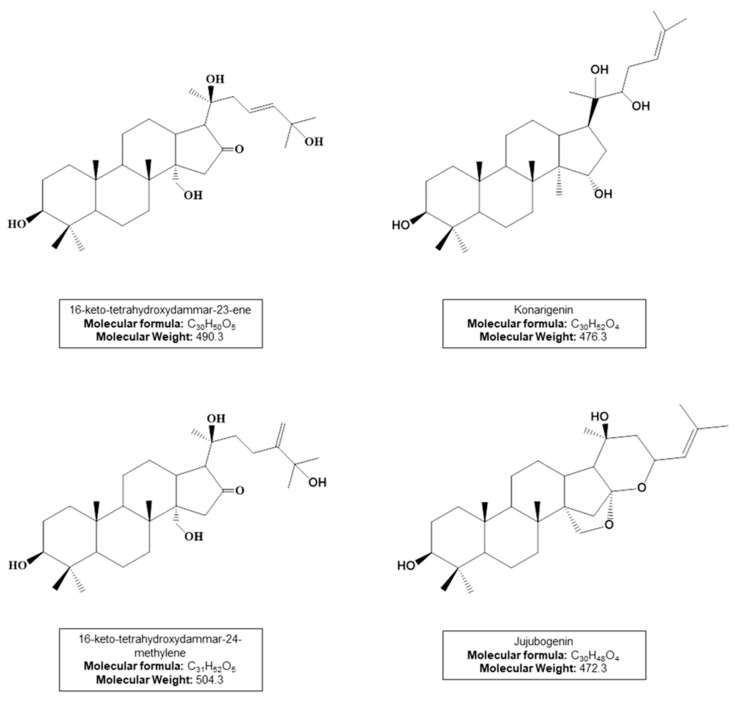
Dammarane-type triterpene aglycones from annotated saponins (compounds **1**–**6**).

The UHPLC-APCI(-)MS/MS analyses show a complex chemical profile and two characteristic regions in the chromatogram, one formed by UV-absorbing compounds (0–5 min), and the other one represented by triterpenes and saponins (10–25 min). Table 1 shows the main saponins annotated in the studied extracts, all of which bear dammarane-type triterpene aglycones (Figure 3), as previously reported for *A. amazonicus* [31,34,35].

Saponin structures were proposed based on the MS fragmentation profile. Compound **1** shows a characteristic MS/MS spectrum of the aglycone 16-keto-tetrahydroxydammar-23-ene, with the loss of 142Da corresponding to its side chain (C_8_H_14_O_2_) (Figure S1). Compound **2** contains an acetyl group attached to the konarigenin aglycone and exhibits characteristic losses of this residue (C_2_H_4_O_2_) (Figure S2). Saponins **3** and **4** bear a C-31 aglycone of the 16-keto-tetrahydroxydammar-24-methylene type, whose fragmentation profiles show losses of C_9_H_16_O_2_ (156Da) corresponding to its side chain (Figures S3 and S4). Finally, compounds **5** and **6** have jujubogenin as the aglycone, showing ions at *m*/*z* 471 [aglycone-H]^−^ or 453 [aglycone-H-H_2_O]^−^ that are products of the precursor ion identified as [M-H]^−^. The Ebelin lactone, *m*/*z* 453 [aglycone-H-H_2_O]^−^, is commonly formed during mass spectrometric analysis of jujubogenin derivatives [35]. This process occurs through common fragmentation reactions in API sources such as APCI. In all MS/MS spectra of the annotated saponins, characteristic neutral losses of sugar residues were observed (162Da for hexoses, 146Da for deoxyhexoses, and 132Da for pentoses).

Contrary to the saponins, the MS profile of the extracts in the region of the UV absorption compounds (0–5 min) is not similar (Figure S7). Preliminary analysis of the λ_max_ of the UV-absorbing peaks suggested that compounds eluted in this zone of the chromatogram could be attributable to a phenolic class of compounds such as flavonoids. In fact, the total phenolic content (TPC) of the most active extract #76 was 46.3 mgEAG/g, confirming the presence of phenols in the extract.

### 2.3. Clean-Up Procedure with Lead Acetate Removes Phenolic Compounds and Promotes Differences in Biological Activity

Taking into account the results of the inhibition rates of the formation of the RBD:ACE2 complex by *A. amazonicus* extracts (Figure 1), and their chemical characterization by mass spectrometry showing a similar saponin profile, a clean-up step on the most active ethanol extract (#76) was performed to separate the UV-absorbing compounds (probably phenolic) from the saponins, aiming to identify whether they could be responsible for the enhanced activity of this extract.

**Figure 4:**
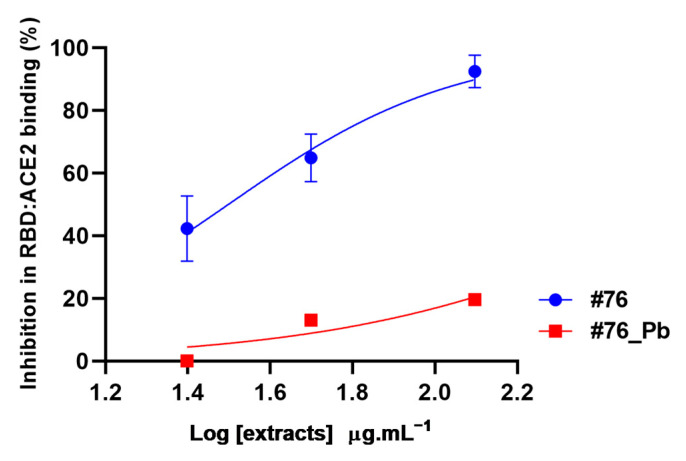
Inhibition of RBD:ACE2 interaction by *A. amazonicus* Ducke bark ethanol extracts before (#76) and after the clean-up step (#76_Pb). #76—Ethanol bark extract, prepared by percolation. #76_Pb—Ethanol bark extract prepared by percolation, after cleaning with lead acetate. These data are the result of two independent duplicates. Multiple *t*-tests between concentrations of each extract showed a significant statistical difference (*p*-value < 0.05). #76 R^2^ = 0.92. #76_Pb R^2^ = 0.81.

Treatment with lead acetate is a classic method used to precipitate phenolic compounds [39], and therefore it was used to clean up the resulting extract from these substances. In sequence, extracts #76 and #76Pb (after clean-up with lead acetate) were evaluated in the RBD:ACE2 complex formation at different concentrations (Figure 4).

The data show that extract #76 interferes in the RBD:ACE2 complex formation in all concentrations tested (25 to 125 μg·mL^−1^), reducing the interaction by at least 40% in a dose-dependent pattern (Figure 4). The highest concentration (125 μg·mL^−1^) reduced the interaction by 92.45 ± 3.65%, which is similar to what was observed at 250 μg·mL^−1^ (Figure 1). Interestingly, the removal of phenolic compounds by lead acetate clean-up abolished the inhibition potential of #76 extract by 70% at the highest concentration (125 μg·mL^−1^), as shown in Figure 4, suggesting that phenolic compounds and not saponins are responsible for hampering RDB and ACE2 interaction.

**Figure 5:**
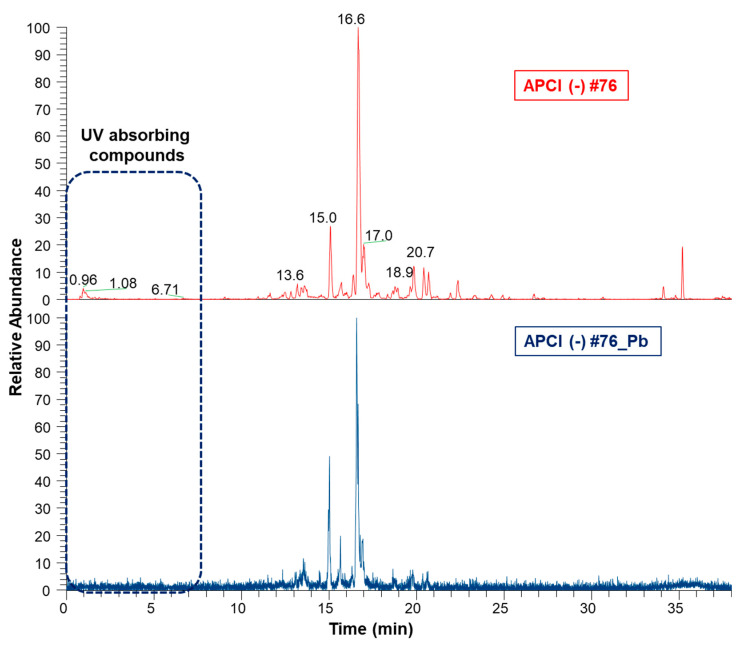
Aligned chromatograms of ethanol extracts before (#76) and after clean-up (#76_Pb) obtained by UHPLC-APCI(-)3D-IT-MS/MS. #76—Ethanol bark extract prepared by percolation. #76_Pb—Ethanol extract bark extract prepared by percolation, after cleaning with lead acetate.

Aiming to compare the chemical profiles of ethanol extracts before and after clean-up, the samples were subjected to ultra-high performance liquid chromatography coupled to tandem MS in the negative ion mode (UHPLC-APCI(-)MS/MS), followed by alignment in MZmine 2.53. Figure 5 clearly shows that the UV-absorbing compounds’ region was simplified upon the clean-up step, indicating the removal of phenolic compounds, the main ones of which were tentatively identified.

UHPLC–PDA–MS/MS analysis of extract #76 revealed the presence of 3′,5′di-*C*-glucosyl phloretin (**8**) and quercetin 3-*O*-deoxyhexoside (**9**) by comparison of their APCI-MS/MS spectrometric data (fragmentation patterns) and photodiode array PDA/UV/Vis with data from the literature (Table 2) [40,41]. Compound (**7**) remained unidentified, but it was possible to hypothesize its structure as a C-glycosyl flavonoid based on the similarity of its mass fragmentation pattern when compared with 3′,5′di-*C*-glucosyl phloretin, (**8**).

**Figure 6:**
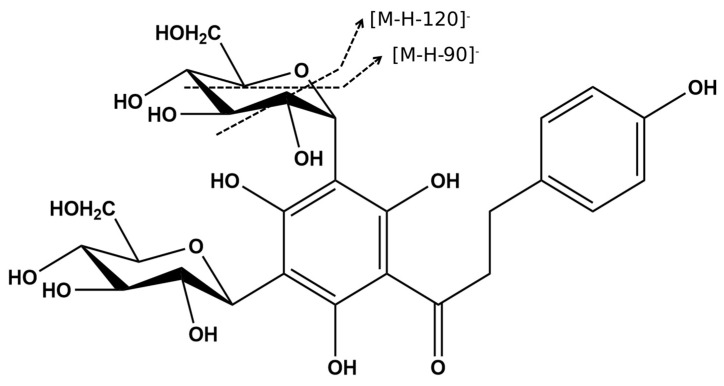
The chemical structure of 3′,5′-di-C-β-glucopyranosyl-phloretin (**8**) and proposed fragmentation of the hexose unit.

The tandem mass spectrum produced by compounds **7** and **8**, showed a pattern typical of a *C*-glycosyl derivative. The fragmentation profiles of these C-glycosylated flavonoids are characterized by the loss of water molecules followed by the fragmentation of the hexose units with fragment ions at *m*/*z* [M-H-90]^−^ and *m*/*z* [M-H-120]^−^ (Figure 6). Compound **7** at *m*/*z* 621 [M-H]^−^ follows this pattern with fragment ions at *m*/*z* 531 [M-H-90]^−^ and *m*/*z* 501 [M-H-120]^−^ [14,42], in addition to the ion at *m*/*z* 603 corresponding to a water loss [M-H-H_2_O]^−^ (Figure S8). This same characteristic fragmentation is observed for compound **8** at *m*/*z* 597 [M-H]^−^, which produces the MS/MS at *m*/*z* 579 [M-H-H_2_O]^−^, *m*/*z* 507 [M-H-90]^−^, and *m*/*z* 477 [M-H-120]^−^ (Figure S9). Compound **8**, which exhibits a λ_max_ at 285 nm, as is typical of the chalcones, was annotated as 3′,5′di-*C*-glucosyl phloretin (Figure 6). The similarity of the mass fragmentation patterns of **7** and **8** suggests the hypothesis that compound **7** is probably a *C*-glucosyl flavonoid derivative.

While compound **8** has been previously described in *Ziziphus spina-christi*, a member of the Rhamnaceae family [40], no data were found in the literature for the mass and fragmentation patterns observed for compound **7**, suggesting that this may be an as yet undescribed *C*-glucosyl flavonoid.

Compound **9**, *m*/*z* 465 [M-H+H_2_O]^−^, has λ_max_ at 255 and 355 nm, which are characteristic of flavonol derivatives [40]. Its MS/MS spectrum shows a fragment ion at *m*/*z* 319 corresponding to the loss of one deoxyhexose unit (146 Da), and a fragment ion at *m*/*z* 301 [M-H-dHex-H_2_O]^−^ (Figure S10), which is in accordance with the mass of a flavonoid aglycone, probably quercetin. In addition, fragment ions characteristic of this aglycone were observed at *m*/*z* 271, 255, and 179 [41].

This is the first report on the phenolic composition of bark extracts of *A. amazonicus*, in which only saponins and triterpenes have been previously identified [31,34,35,37,38]. Despite saponins being the major compounds in the plant extracts, the phenolic compounds described in this study seem to be responsible for the inhibition of SP and ACE2 interaction.

Polyphenolic compounds are among the most promising chemical class of SARS-CoV-2 inhibitors [43]. A recent review compiled the structure–activity relationships of plant phenolics with antiviral activities against human coronaviruses [44], showing that data on viral entry inhibitors are scarce. The flavonoid luteolin has been shown to inhibit the entry of HIV-luc/SARS pseudotyped virus and wild-typed SARS-CoV into Vero E6 cells without substantial cytotoxicity. Emodin, an anthraquinone, inhibited the interaction between SARS-CoV S protein and ACE2 receptor in a dose-dependent manner.

However, most of the studies with SARS-CoV-2 SP and ACE2 interactions are in silico studies. Computational studies showed that catechin and curcumin bind the interface of the ‘RBD:ACE2-complex’, suggesting the efficacy of these two polyphenols in hindering the formation of SP and ACE2 complex [45]. Structure-based virtual screening of the Taiwan Database of Extracts and Compounds identified 39 natural products targeting the viral receptor-binding domain (RBD) of the SARS-CoV-2 spike protein; among them, amentoflavone was selected as the best one [46]. Recently, surface plasmon resonance of candidate molecular binders has been performed to determine the binding affinities and kinetics for RBD of a group of five compounds previously selected by a virtual screening of a natural product database, one of which was 5,4′-di-hydroxy-6,7-di-glucosyl-flavanone [47].

Concerning chalcones, a recent in silico study screened a library of natural and synthetic chalcones against homology-modeled S protein. Analysis of protein–ligand docking revealed that phloretin can interact with the spike proteins’ key RBD, pointing to this class of natural compounds as promising [48].

Interestingly, in another recent in silico study, phloretin showed a strong inhibitory effect on the crucial residues ASN417, SER496, TYR501, and HIS505 of the omicron receptor-binding domain (RBD), which are supported for the inviolable omicron and angiotensin-converting enzyme II (ACE2) receptor interaction [49]. Additionally, the authors state that phloretin is suggested as the antiviral drug against omicron B.1.1.529, which strongly supports our findings that 3′,5′di-*C*-glucosyl phloretin is in part responsible for blocking the SP and ACE2 interaction in extract #76.

On the other side, flavones such as quercetin and their derivatives are currently considered promising compounds for COVID-19 treatment [50]. Quercetin possesses a 15-carbon skeleton with a chromone core comprising bicyclic 1,4-benzopyrone (A- and C- rings) substituted on carbon 2 with a catechol moiety (B-ring), with hydroxyl groups at positions 3′, and 4′ with electron-donating activity [51]. The presence of hydroxyl groups in the B-ring reflects positively on its binding affinities to the viral target [52]. In silico and in vitro studies have been focused on quercetin, testing it experimentally against some targets such the spike protein of SARS-CoV-2, with promising results showing its potential as an antiviral agent [53,54].

In an in silico study [55], the authors suggest that beyond the interaction with active sites of the spike protein of SARS-CoV-2, polyphenols from *Geranii herba* could interact in the cell surface receptor glucose-regulated protein 78 (GRP78) to regulate the cell signaling to release ER stress and other processes. They could also interact either directly or indirectly with the Cys/His dyad of the main protease to inhibit viral replication. In this way, polyphenols could interfere with viral pathogenesis in various stages [56].

Moreover, flavonoids are known for their potential ability to regulate inflammatory mediators and inhibit endothelial activation, among other factors, which might be beneficial in regulating the cytokine storm during SARS-CoV-2 infection, and may positively influence inflammatory changes associated with COVID-19 [57]. Additionally, phenol-rich extracts from plants have also demonstrated antiinflamatory activity [58,59].

The results presented here show evidence that supports that compounds belonging to chalcones and flavones in the *A. amazonicus* extracts were able to inhibit the binding of the viral spike protein to its ACE2 receptor, which is one vital mechanism for the entry and replication of SARS-CoV-2 viruses.

### 2.4. The Characterized Extracts Can Inhibit SARS-CoV-2 In Vitro Replication

Since plant extracts represent complex matrices composed of a wide range of substances, it is possible that the same extract targets different steps in the virus life-cycle, which results in distinguishable antiviral activity. Considering the RBD:ACE2 inhibition promoted by *A. amazonicus* extracts, we assessed whether they would be able to act at a different stage of infection. For this purpose, the Calu-3 cells, a well-established model for SARS-CoV-2 in vitro experimental infection, were used to evaluate the cytotoxicity and antiviral activity of all plant extracts [60,61].

**Figure 7:**
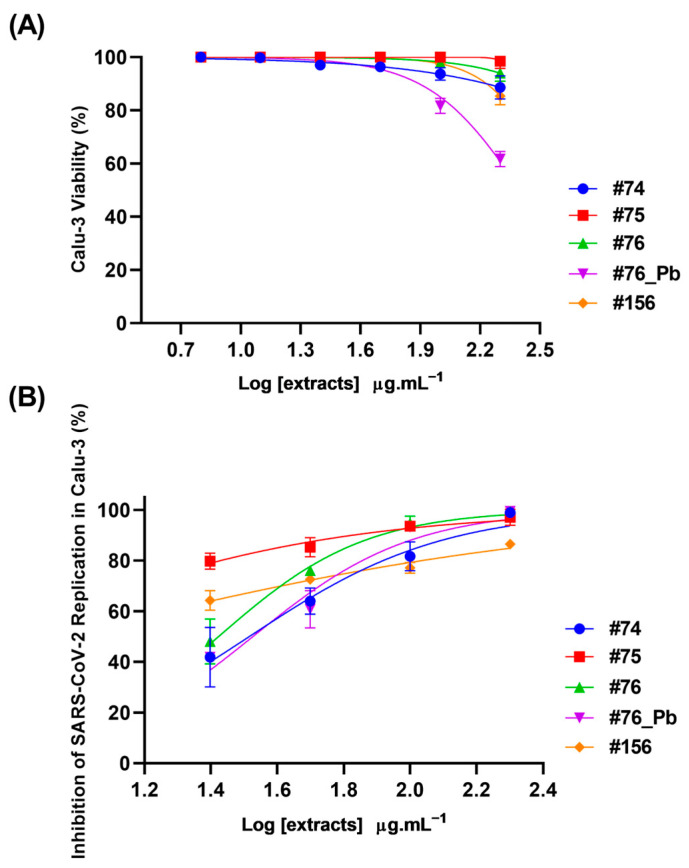
Cytotoxicity and antiviral effects of extracts from *Ampelozizyphus amazonicus*. Calu-3 cells were exposed to different concentrations (6.3, 12.5, 25, 50, 100 and 200 µg·mL^−1^) of extracts for 72 h at 37 °C and 5% CO_2_. The cell viability was determined by MTT assay (**A**). Calu-3 cells were infected with SARS-CoV-2 (MOI 0.01) for 1 h, then the medium was changed to a medium with extracts in different concentrations (25, 50, 100 and 200 µg·mL^−1^). The supernatants were harvested 24 at hpi at 37 °C and 5% CO_2_ for virus titration by a Plaque-Forming Units (PFU/mL) assay (**B**). Extracts R^2^ varied from 0.85 to 0.97.

Although saponins are known to be potential cytotoxic agents [62], the cell toxicity determined by MTT assay showed that none of the uncleaned extracts were toxic to Calu-3 cells at the highest concentration tested (200 µg·mL^−1^). The only exception was #76_Pb, which reduced cell viability by 40% at the highest concentration (Figure 7A). Despite this, all the extracts exhibited a concentration required to reduce cell viability by 50%, with values (CC_50_) higher than 200 µg·mL^−1^.

The antiviral activity of the extracts was determined by the reduction in SARS-CoV-2 replication in Calu-3 cells. All the extracts were able to inhibit SARS-CoV-2 replication at 24 h (Figure 7B). The concentration required to inhibit viral infection-induced cytopathogenicity by half was most promising for the extracts #75 and #156, which presented EC_50_ values < 25 µg·mL^−1^ (Table 3).

According to the chromatographic profile shown in Figure 2, the samples seem to be very similar regarding the saponins profile, which may explain the fact that they are all active in inhibiting SARS-CoV-2 replication. Although the results obtained previously demonstrate that *A. amazonicus* extracts can act on the ligand–receptor interaction of a structural protein and that this action is linked to the phenolic compounds in the extract, antiviral activity is a complex process and can exhibit different possibilities of interference.

In this section, the results shown reflect that *A. amazonicus* extracts (especially #75 and #156) also act on a distinct target, quite possibly non-structural proteins, since the model represents a scenario in which the infection is already established. RBD:ACE2 binding assays demonstrate that saponins are not important for the interaction’s inhibition, unlike the polyphenols in the extract. However, it is known that saponins have anti-inflammatory and immunomodulatory effects, properties already demonstrated for these same extracts and that are important in the clinical approach to COVID-19 treatment [30,31,63,64]. Additionally, this action would also support the traditional use of this species by Amazonian communities [32]. Interestingly, there is one record for an anti-SARS-CoV-2 compound in the Rhamnnaceae family: jubanine B, a cyclopeptide alkaloid from *Ziziphus spina-christii* (L) Desf. was demonstrated to be a promising ligand of 3CL^pro^ protein, the main protease of the SARS-CoV-2 virus [65].

*A. amazonicus* is an Amazonian adaptogen [29], and plants with this characteristic could consist of interesting models to be explored in drug discovery against SARS-CoV-2. Several traditional Chinese medicine protocols have been evaluated since 2020 in the treatment of COVID-19, using in silico, in vivo, and in vitro approaches [66]. As the basis of these practices are contained in medicinal plants and natural products, the good results presented here show that alternatives from plant secondary metabolites are very promising.

*Panax ginseng* Meyer, popularly known as Korean ginseng, is a plant with a high content of saponins structurally similar to the ones present in *A. amazonicus*, with dammarane triterpene structure [67]. It has been shown, in vivo that transgenic mice that were fed with ginseng extracts had much lower virus titers than the ones that were not. Additionally, interferon-gamma (IFN-γ) production was significantly higher in the lung tissue of the mice fed with ginseng extracts [68], demonstrating the potential of this species in SARS-CoV-2 infection, not only in virus replication but also in host response. Similarly to *A. amazonicus*, *P. ginseng* is also used in traditional medicine as an immune system fortifier, and has been pointed out as a potential treatment for COVID-19 [69]. Therefore, *A. amazonicus* is an interesting species to be considered in new drug discoveries to counter SARS-CoV-2, since it presents promising substances with proven action in its replication process.

## 3. Materials and Methods

### 3.1. Chemicals, Materials and Plant Extracts

Lead acetate trihydrate crystal reagent (Spectrum^®^, Chemical MFG Corp., Gardenia, CA 90248, New Brunswick, NJ, USA), absolute ethanol 96% (EtilRio^®^/p.a.), acetonitrile (HPLC/spectrum grade), and formic acid (LC-MS grade) were from Tedia (Fairfield, CA, USA). Water (18.2 MΩ∙cm) was from a Millipore Direct-Q^®^ purification system.

Four different *A. amazonicus* bark extracts from our biobank library (registered at the Sistema Nacional de Gestão do Patrimônio Genético e do Conhecimento Tradicional Associado, SISGEN, under the registration number CB8C853), coded with numbers #74, #75, #76 and #156, were used for in vitro assays.

*Ampelozizyphus amazonicus* was collected in August 2008, in the Brazilian Amazon region of Oriximina (Para state). Bark was dried in a ventilated oven (Marconi, model MA037) and ground in a hammer mill (Marconi, model MA340, serial 9304176). The preparation of extracts #74, #76 and #156 is reported in the literature (#74) [30], (#156) [31] and (#76) [34].

In brief, extract #74 was prepared with 250 g of dried and ground bark submitted to extraction with boiling water (5% *w*/*v*) for 15 min and filtered. A second extraction was performed with boiling water (2.5%, *w*/*v*, 30 min). The extracts were mixed and infused into a spray-dryer nozzle unit of a Buchi ¨Mini Spray Dryer B-290 (Buchi Laboratorius-Technik AG, Flawil 1, Switzerland) [30]. The extractive yield was 4.5%. Extract #75 was prepared with commercial ethanol (96%) from the cake resulting from the aqueous extraction that generated extract #74, until exhaustion of plant material. It was then filtered and concentrated under reduced pressure in a rotary evaporator, at a temperature not exceeding 40 °C. The extractive yield was 7.3%. For extract #76, ground and dried bark (346.544 g) were extracted with commercial ethanol (96%) by percolation until exhaustion. The extract was filtered, and ethanol was removed by rotary evaporation at 40 ◦C under reduced pressure [34]. The extractive yield was 8.61%. Finally, to extract #156, dried and ground bark was used for the preparation of extract at the Centroflora Group facility, Botucatu, SP, Brazil. The bark (14.9 kg) was submitted to a two-step extraction process as follows: extraction with boiling water (5% *w*/*v*) for 15 min, followed by a second extraction with boiling water (2.5%, *w*/*v*) for 30 min. The extracts were mixed and filtered (using a rotary drum filter with a mesh of 30 μm) and then concentrated until the total solid level reached 24% (*w*/*v*) (Bernauer evaporator). Final drying was achieved by spray drying as reported in [31]. The extractive yield was 4.52%.

A brief description of the methodologies is presented in Table 4. The work was authorized by Conselho de Gestão do Patrimônio Genético (CGEN) to access the traditional knowledge with bioprospecting purposes, by the Resolution CGEN no. 213 (6 December 2007), renewed in the Resolution CGEN no. 87/2012, and by the authorizations AEAC3E9 in 2018, and A5A49CA in 2022.

### 3.2. Clean-Up Procedure with Lead Acetate

Separately, 0.5 g of #76 was mixed with 20 mL of 50% ethanol (EtOH) solution and vortexed for 15 min. Then, 10 mL of 10% lead acetate was added in each tube and vortexed for 25 min at 50 °C. After centrifugation, the supernatant was collected, lyophilized, and coded as #76_Pb.

### 3.3. Sample Preparation for Chemical Investigation

Separately, 10 mg of each sample (#76, and #76_Pb) was transferred to 2 mL microtubes. Then, 1 mL of acetonitrile (ACN) was added to each microtube, sonicated for 15 min and vortexed for 30 s. After that, 200 µL of each solution was transferred to a 1.5 mL microtube and then 300 µL of ACN and 500 µL of purified water were added. The final solution (1:1, *v*:*v*) was vortexed for 30 s and centrifuged at 10,000 RPM for 15 min. 750 µL of supernatants were injected and analyzed.

### 3.4. Sample Preparation for RBD:ACE2 Inhibition Assays

Separately, 1 mg of each sample was transferred to a microtube and solubilized in 20 µL of dimethyl sulfoxide (DMSO—Sigma-Aldrich, St. Louis, MO, USA) and 80 µL ultrapure water. The solutions were vortexed for 1 min and then centrifuged (5804 R—Eppendorf) for 5 min at 10,000 RPM. After, the supernatant of each solution was collected, identified, and refrigerated.

### 3.5. LC-DAD-APCI-MS/MS Analysis

Ultra-high-performance liquid chromatography with an ultraviolet detector coupled to tandem mass spectrometry (UHPLC-UV-MS/MS) analyses was performed using a UHPLC Dionex^TM^ UltiMate^TM^ 3000 system, coupled to an LCQ Fleet (ThermoFisher Scientific, Waltham, MA, USA) consisting of an oven, a solvent degasser, an ultra-high-pressure pump, an autosampler, a diode array detector and a column temperature manager. An ACQUITY UPLC^®^ BEH C18 reversed-phase column (2.1 × 100 mm, 1.7 µm, Waters) was used at a flow rate of 0.35 mL·min^−1^. The column temperature was kept at 30 °C, and the mobile phases were 0.1% formic acid in water for A, and acetonitrile for B. The gradient elution mode was as follows: 25% B in 0–2 min, 25–50% in 2–30 min, 50–98% in 30–31 min, 98–98% in 31–33 min, 98–25% in 33–35 min, and 25–25% in 35–38 min. Wavelength channels used in the DAD instrument were 210 nm, 254 nm, 275 nm, and 360 nm with a bandwidth of 2 nm.

The mass spectrometer (MS), equipped with an atmospheric pressure chemical ionization (APCI) source and an ion trap analyser (with 1.000 of resolution), was operated in negative ion mode. High-purity nitrogen (N_2_) was used as the sheath gas (30 arbitrary units) and auxiliary gas (15 arbitrary units). High-purity helium (He) was used as the collision gas. The MS parameters used were 6.0 kV of source voltage, −2 V of capillary voltage, −116 V of tube lens voltage, 400 °C of capillary temperature and 400 °C of APCI vaporizer temperature. Full scan data acquisition (mass range: *m*/*z* 100–1500) and data-dependent acquisition (topN = 3) were performed. The normalized collision energy of the collision-induced dissociation (CID) cell was set at 35 eV. Data obtained from LC–MS analyses were converted to mzML format using the Proteowizard software. Then, the data were processed in Mzmine 2.53.

### 3.6. Total Phenolic Content (TPC)

The phenolic content of the most active extract (#76) was assessed as previously described [70]. Briefly, 5 mg of the ethanol extract was diluted in 1 mL of commercial ethanol. Then, an aliquot of 200 μL of this solution was transferred to a new microtube with 800 μL of ultrapure water. The 1 mg/mL solution was used for quantification. In a flat-bottom 96-well plate, 0.1 mL aliquots were added to 0.5 mL of Folin–Ciocateu reagent (10% *w*/*v*). The calibration curve was constructed using gallic acid ranging from 0.5 mg/mL to 0.003 mg/mL. The sample was tested in 500 and 250 μg·mL^−1^. The total phenolic content of the extract was expressed in milligrams of gallic acid equivalent per gram of extract (mg GAE/g).

### 3.7. Lumit™ RBD:ACE2 Interaction Assays

To assess if *A. amazonicus* extracts would have any potential in blocking SARS-CoV-2 entry into cells, we used the commercial kit Lumit™ SARS-CoV-2 spike RBD:ACE2 immunoassay (Promega), following the instructions of the manufacturer. Lumit™ methodology relies on the use of the RBD (receptor-binding domain) portion of the SARS-CoV-2 spike protein, as the one responsible for recognizing and binding to the ACE2 receptor. Briefly, samples were added to a white, flat bottom, 96-well plate in a concentration of 250 μg·mL^−1^ (80% ultrapure water, 20% dimethylsulfoxide), followed by the addition of RBD and ACE2 reagents (7.5 nM). Detection is performed by secondary antibodies coupled to light particles and the substrate for this reaction. The binding of the antibodies to proteins promotes the reconstitution of NanoBiT particles, a technology developed by Promega, generating bioluminescence which was recorded in a SpectraMax M5 (Molecular Devices) microplate reader.

### 3.8. Calu-3 Cytotoxicity Assay

Calu-3 (a submucosal gland cell line, generated from a bronchial adenocarcinoma and kindly donated by the Farmanguinhos platform RPT11M) cells (1.5 × 10^4^ cell/well) were incubated in 96-well plates with the extracts #74, #75, #76, #76_Pb and #156 in different concentrations (6.3, 12.5, 25, 50, 100 and 200 µg·mL^−1^) for 72h at 37 °C, 5% CO_2_. Afterward, cell viability was determined by MTT assay, according to the manufacturer’s instructions. Briefly, 5 mg·mL^−1^ of 3-(4,5-dimethylthiazol-2-yl)-2,5-diphenyltetrazolium bromide (MTT, Sigma) in 1× PBS was added to monolayers of cells for 2 h at 37 °C, 5% CO_2_. Then, 10% SDS was added, and plates were read in spectrophotometer at 570 nm. All the compounds were resuspended in 100% dimethyl sulfoxide (DMSO) for the in vitro tests. The DMSO final concentrations do not exceed 1% (*v*/*v*) in the experiments, thereby not affecting the cells growth.

### 3.9. Inhibition of SARS-CoV-2 Replication in Calu-3 Cells

Monolayers of Calu-3 cells (1.5 × 10^4^ cell/well) cultured in 96-well plates were infected with SARS-CoV-2 B.1 lineage isolate (GenBank MT710714, SisGen AC58AE2) in multiplicity of infection (MOI) 0.01 for 1 h at 37 °C, 5% CO_2_. After this period, the cells were treated with the extracts #74, #75, #76, ##76_Pb and #156 at concentration curve (25, 50, 100 and 200 µg·mL^−1^) for 24 h. Afterward, the supernatants were harvested and the virus titrated by plaque-forming units (PFU/mL) to determine viral growth.

For the PFU/mL assay, the monolayers of Vero E6 (African green monkey kidney, ATCC CRL-1586) cells (1.5 × 10^4^ cell/well) were exposed to different dilutions of viral supernatants (1:200–1:25,600) for 1h at 37 °C, 5% CO_2_. Then, the same well volume of carboxymethylcellulose 2.4% medium (DMEM-HG 10×, 2.4 carboxymethylcellulose and 2% fetal bovine serum) was added, and the cells were incubated at 37 °C, 5% CO_2_ for 72 h. After this, the cells were fixed with formalin 10% and stained with crystal violet 0.04%, and the quantification of PFUs was carried out to determine the virus titer. All experimental procedures were executed at a biosafety level 3 (BSL3) multi-user facility, according to WHO guidelines [71].

### 3.10. Statistical Analysis

Graphs were generated using the GraphPad Prism 8.0 software. Values of EC_50_ were determined by non-linear regression of log (inhibitor) vs. normalized response. Values correspond to the best curve generated based on R^2^ values ≥ 0.9. All experiments were realized with three technical replicates.

## 4. Conclusions

Until now, all studies regarding *A. amazonicus* have attributed the species’ promising pharmacological potential to saponins, its class of major compounds. This work is the first to report the phenolic composition of *A. amazonicus* and to show the important role that these substances hold in inhibiting the binding between SARS-CoV-2 spike protein and ACE2 receptor. However, when evaluating a different target, such as viral replication post-infection, saracura-mirá extracts also present promising activity reducing viral titers, which means that saponins may also contribute to the antiviral potential of *A. amazonicus*. In addition, the anti-inflammatory and immunomodulatory activity of these extracts has already been proven and attributed to this class of compounds. These two features represent very important approaches to the treatment of SARS-CoV-2 infection, as they are able to control symptoms and prevent evolution into severe cases, as has been highlighted in the popular use of *A. amazonicus*. Therefore, it is believed that *A. amazonicus* can contribute to controlling the infection caused by SARS-CoV-2 through several mechanisms, standing out as a multitarget agent. The data presented here, together with the literature and recent reports on the medicinal use of *A. amazonicus*, strongly suggest that this species could be a possible candidate and model for the development of new drugs to treat COVID-19.

## Figures and Tables

**Table 1:** Main saponins annotated in the *A. amazonicus* aqueous and ethanol bark extracts.

Compound	R_t_ (min)	[M-H]^−^ *m*/*z*	Molecular Formula	MS^2^	Aglycone	Sugar Residue ^a^
**1**	13.6	959.4	C_48_H_80_O_19_	941 (-H_2_O), 817 (-C_8_H_14_O_2_), 797 (-Hex), 779 (-H_2_O-Hex), 655 (-C_8_H_14_O_2_-Hex), 509 (-C_8_H_14_0_2_-Hex-dHex)	16-keto-tetrahydroxydammar-23-ene	2Hex, 1dHex
**2**	15.0	973.3	C_49_H_82_O_19_	913 (-C_2_H_4_O_2_), 841 (-Pen), 811 (-Hex), 781 (-C_2_H_4_O_2_-Pen), 751 (C_2_H_4_O_2_-Pen), 619 (C_2_H_4_O_2_-Pen-Hex)	konarigenin	2Hex, 1Pen
**3**	16.6	943.4	C_48_H_80_O_18_	811 (-Pen), 781 (-Hex), 763 (-H_2_O-Hex), 649 (’Hex-Pen), 479 (-C_9_H_16_O_2_-Hex-d-Hex), 347 (-C_9_H_16_O_2_-Hex-dHex-Pen)	16-keto-tetrahydroxydammar-24-methylene	1Hex, 1dHex, 1Pen
**4**	17.0	957.3	C_49_H_82_O_18_	801 (-C_9_H_16_O_2_) 811 (-dHex), 795 (-Hex), 649 (-Hex-dHex)	16-keto-tetrahydroxydammar-24-methylene	2dHex, 1Hex
**5**	18.9	1059.3	C_52_H_84_O_22_	927 (-Pen), 897 (-Hex), 879 (-H_2_O-hex), 756 (-Hex-Pen), 735 (-2Hex), 603 (-2Hex-Pen), 453 (-H_2_O-2Hex-2Pen)	jujubogenin	2Hex, 2Pen
**6**	20.7	897.5	C_46_H_74_O_17_	765 (-Pen), 735 (-Hex), 603 (-Pen-Hex), 471 (-Hex-2Pen)	jujubogenin	1Hex, 2Pen

^a^: Hex: hexose; dHex: deoxyhexose; Pen: pentose (sugar residues attached to the aglycone skeleton).

**Table 2:** Main phenolic compounds annotated by UHPLC-APCI-MS/MS in bark ethanol extract (#76) before the clean-up step.

Compound	Class	Compound Molecular Formula	Rt (min)	[M-H]^−^ *m*/*z*	MS^2^	λ_max_	Reference
**7**	flavonoid	*C*-glycosylated flavonoid (UI) *	0.96	621.4	603 (M-H-H_2_O), 531 (M-H-90), 501 (M-H-120) 441 2x (M-H-90), 381 2x (M-H-120)	295	-
**8**	chalcone	3′,5′di-*C*-glucosyl phloretin C_27_H_34_O_15_	0.96	597.5	579 (M-H-H_2_O), 507 (M-H-90), 477 (M-H-120) 417 2x (M-H-90), 357 2x (M-H-120)	285	[40]
**9**	flavonol	quercetin 3-*O*-deoxyhexoside C_21_H_20_O_11_	0.98	465.7 [M-H +H_2_O]^−^ adduct	319 (-dHex), 301 (-dHex-H_2_O) 283 (-dHex-2H_2_O) 255 (-dHex-2H_2_O -CO)	255, 355	[41]

* UI: Unidentified.

**Table 3:** Extracts EC_50_ (µg·mL^−1^) values.

	Time Post-Infection (h)	#74	#75	#76	#76_Pb	#156
EC_50_ _(_µg·mL^−1^_)_	24	32.79 ± 3.4	<25.0	26.50 ± 1.3	33.61 ± 3.2	<25.0

**Table 4:** Code number and extract preparation methods for each *A. amazonicus* extracts.

Code Number	Code	Extraction Preparation Method	Ref.
#74	SART	Aqueous bark extract prepared in the laboratory scale according to the traditional beverage	[30]
#75	SET-MT	Ethanol bark extract prepared after the aqueous extraction of extract #74	-
#76	SETMA	Ethanol bark extract prepared by percolation	[34]
#156	SARFLORA	Aqueous bark extract prepared in a pilot-scale industrial plant, according to the traditional method	[31]

## Data Availability

Not applicable.

## Supplementary Materials

The following supporting information can be downloaded at: https://www.mdpi.com/article/10.3390/molecules28073159/s1, Figure S1: MS/MS spectra of compound **1** at *m*/*z* 959.4 [M-H]^−^; Figure S2: MS/MS spectra of compound **2** at *m*/*z* 973.3 [M-H]^−^; Figure S3: MS/MS spectra of compound **3** at *m*/*z* 943.4 [M-H]^−^; Figure S4: MS/MS spectra of compound **4** at *m*/*z* 957.3 [M-H]^−^; Figure S5: MS/MS spectra of compound **5** at *m*/*z* 1059.3 [M-H]^−^; Figure S6: MS/MS spectra of compound **6** at *m*/*z* 897.5 [M-H]^−^; Figure S7: MS profile of extracts in the region of UV absorption compounds (0–5 min); Figure S8: MS/MS spectra of compound **7** at *m*/*z* 621.4 [M-H]^−^; Figure S9: MS/MS spectra of compound **8** at *m*/*z* 597.5 [M-H]^−^; Figure S10: MS/MS spectra of compound **9** at *m*/*z* 465.7 [M-H+H_2_O]^−^ (water adduct).

## Abbreviations

ACE2	Angiotensin-converting Enzyme 2
RBD	Receptor Binding Domain
ACN	Acetonitrile
APCI	Atmospheric Pressure Chemical Ionization
CC_50_	Half-maximal Cytotoxic Concentration
CGEN	Conselho de Gestão do Patrimônio Genético
CID	Collision-induced Dissociation
COVID-19	Coronavirus Disease 2019
DAD	Diode Array Detector
DMSO	Dimethyl Sulfoxide
EC_50_	Half-maximal Effective Concentration
EtOH	Ethanol
FRET	Fluorescence Resonance Energy Transfer
LC	Liquid Chromatography
MS	Mass Spectrometry
MTT	3-[4,5-dimethylthiazol-2-yl]-2,5 diphenyl tetrazolium bromide
ORF	Open Reading Frame
RBD	Receptor Binding Domain
R_t_	Retention time
SARS-CoV-2	Severe Acute Respiratory Syndrome Coronavirus 2
SP	Spike Protein
UHPLC	Ultra High-Performance Liquid Chromatography
WHO	World Health Organization
