# Supplementary material for: Anti-SARS-CoV-2 Activity of Ampelozizyphus amazonicus (Saracura-Mirá): Focus on the Modulation of the Spike-ACE2 Interaction by Chemically Characterized Bark Extracts by LC-DAD-APCI-MS/MS
Source: Molecules. 2023 Apr 1;28(7):3159. doi: 10.3390/molecules28073159 (PMC10095690; doi:10.3390/molecules28073159)

Supplementary Material

Anti-SARS-CoV-2 Activity of *Ampelozizyphus amazonicus* (Saracura-Mirá): Focus on the Modulation of the Spike-ACE2 Interaction by Chemically Characterized Bark Extracts by LC-DAD-APCI-MS/MS

Figure S1: MS/MS spectra of compound **1** at *m/z* 959.4 [M-H]$^-$

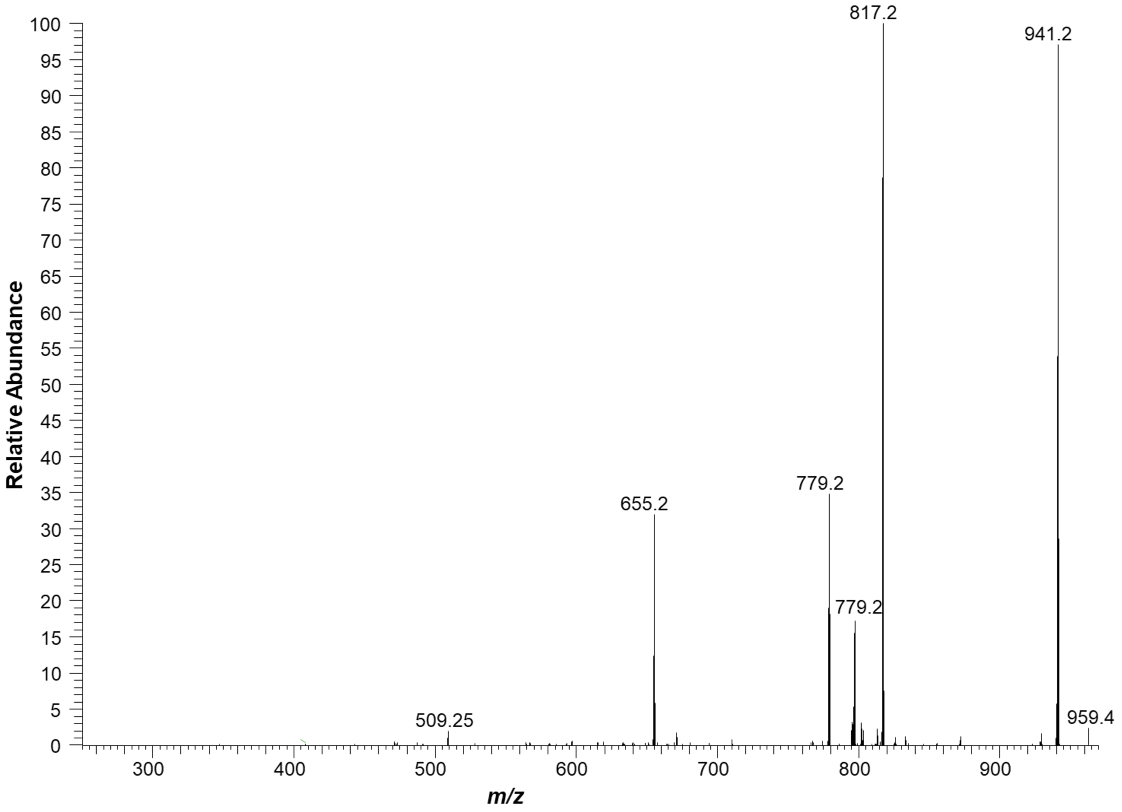

Figure S2: MS/MS spectra of compound **2** at *m/z* 973.3 [M-H]$^-$

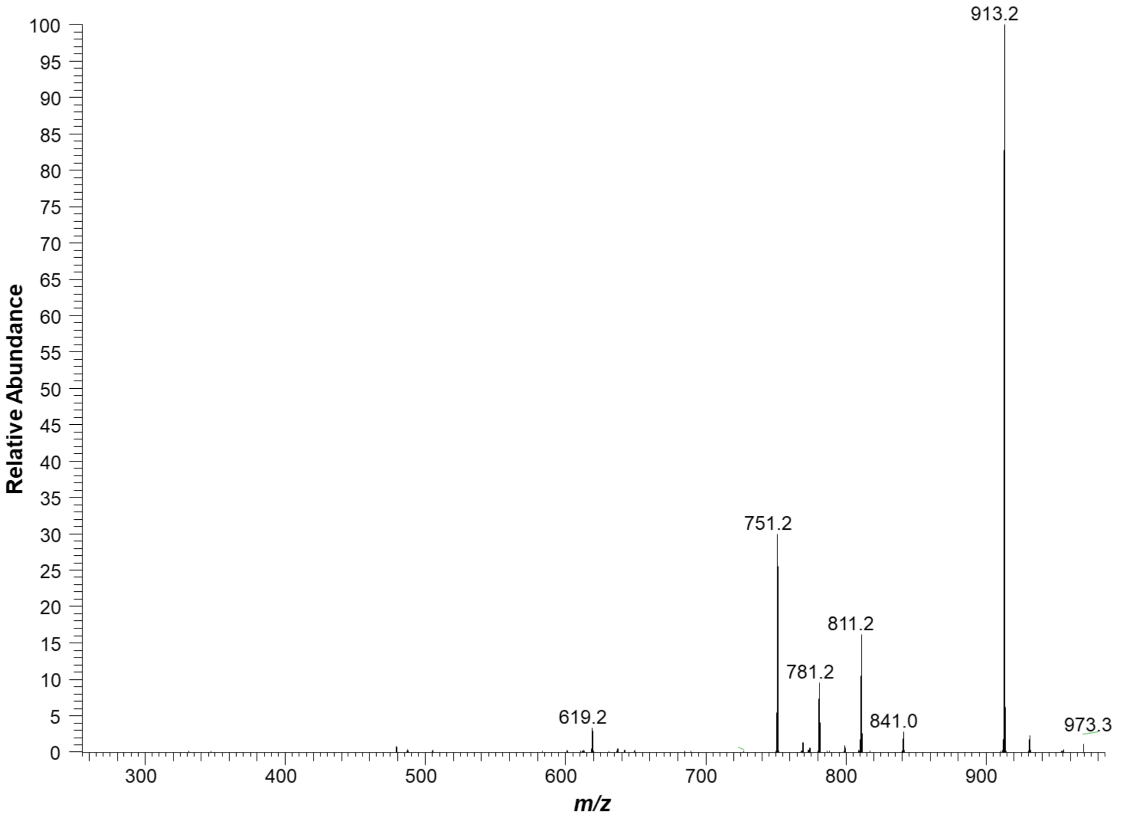

Figure S3: MS/MS spectra of compound **3** at *m/z* 943.4 [M-H]$^-$

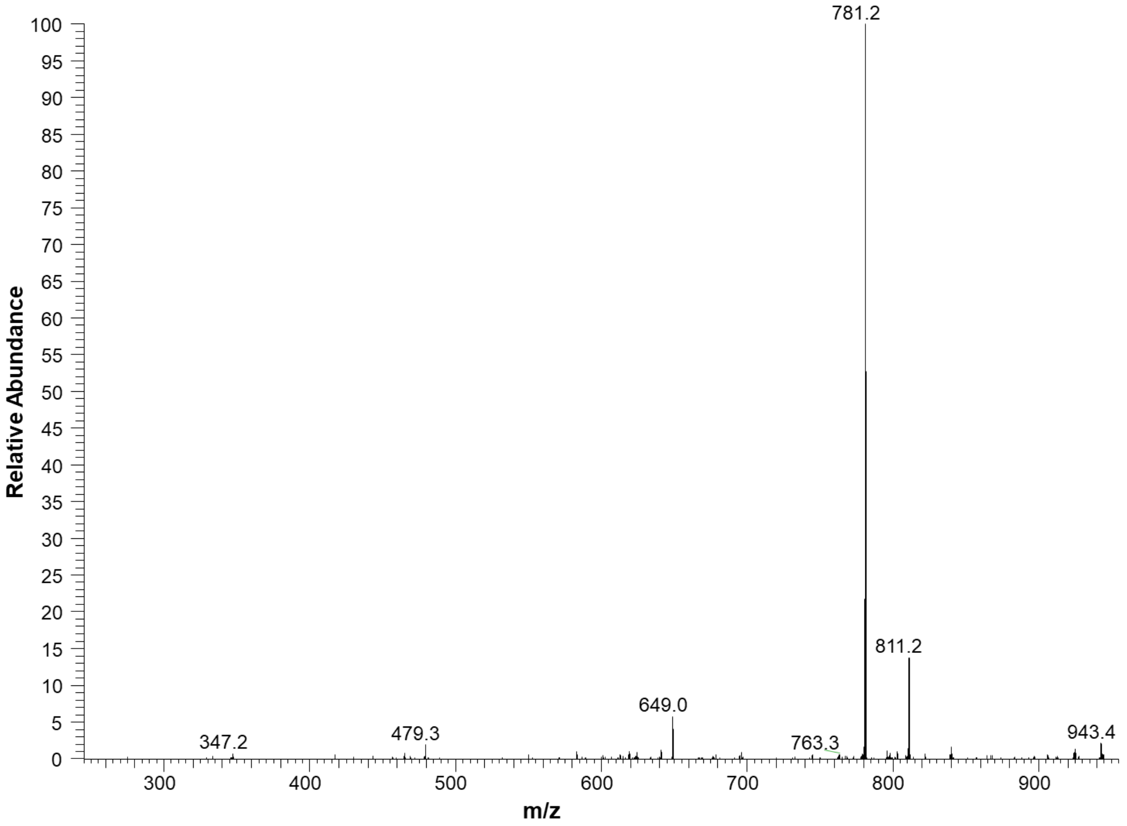

Figure S4: MS/MS spectra of compound **4** at *m/z* 957.3 [M-H]$^-$

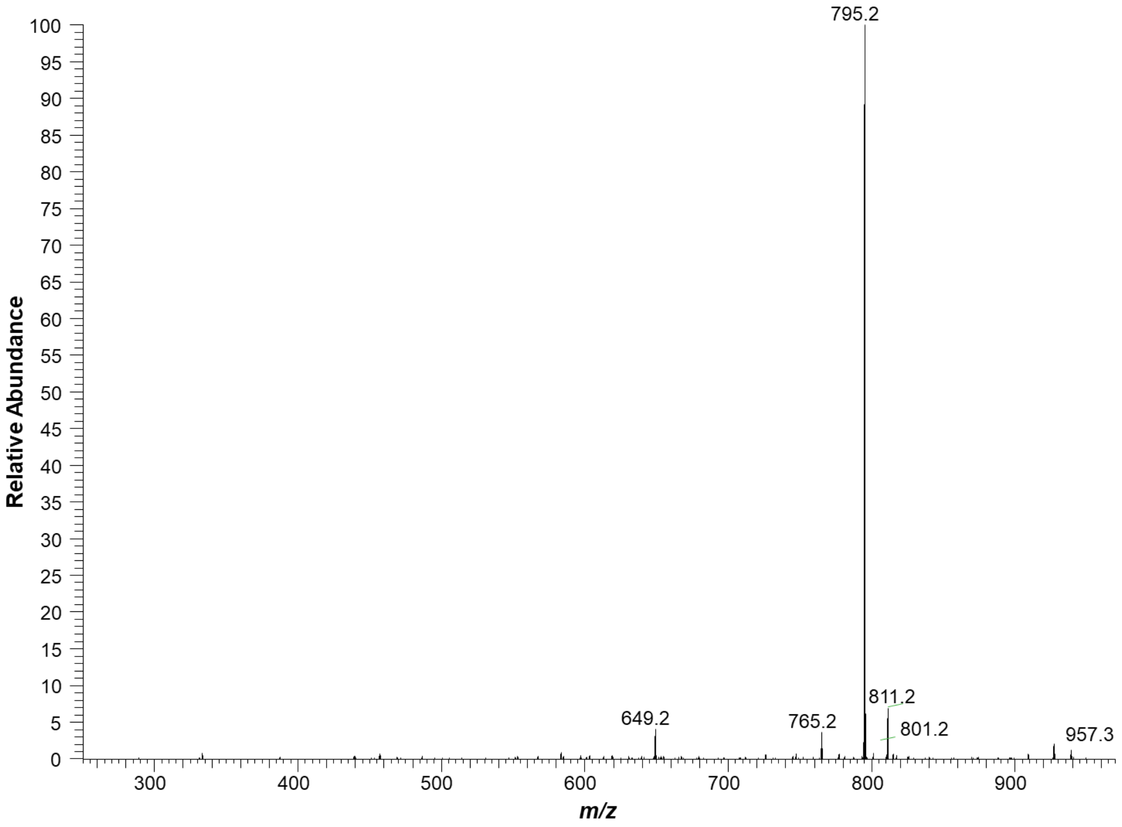

Figure S5: MS/MS spectra of compound **5** at *m/z* 1059.3 [M-H]$^-$

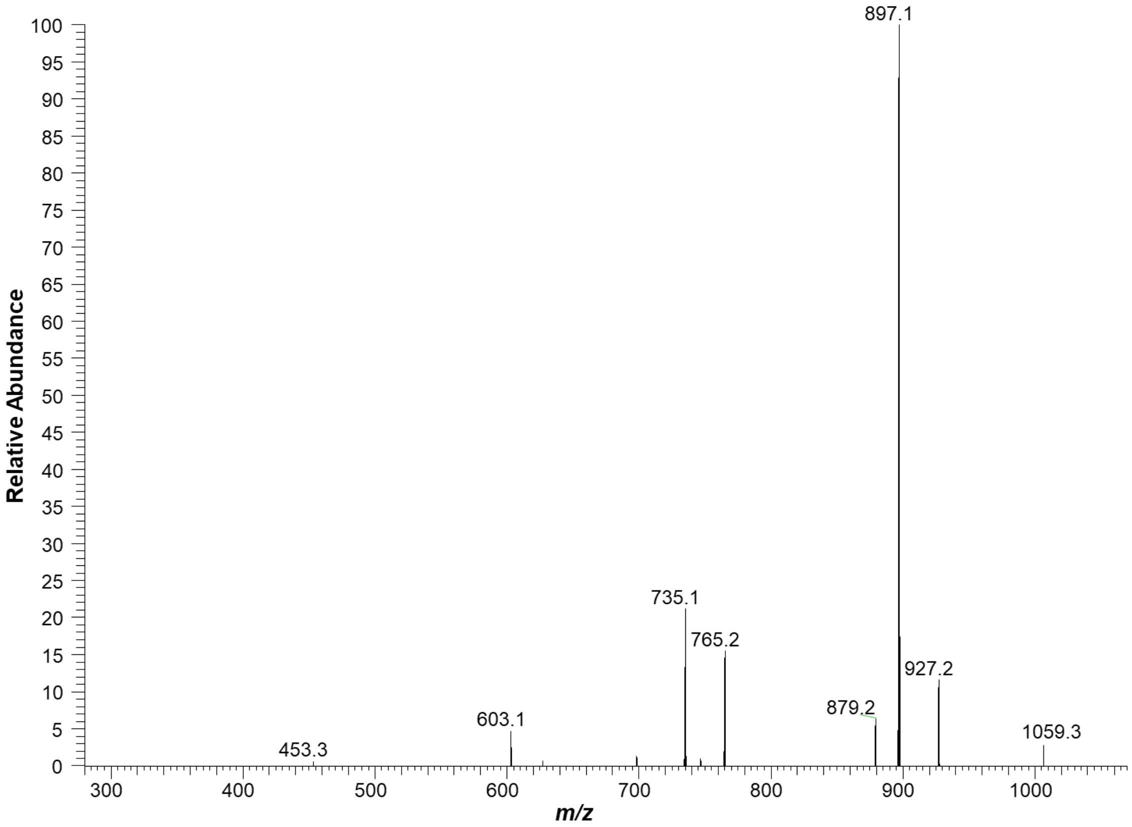

Figure S6: MS/MS spectra of compound **6** at *m/z* 897.5 $[M-H]^-$

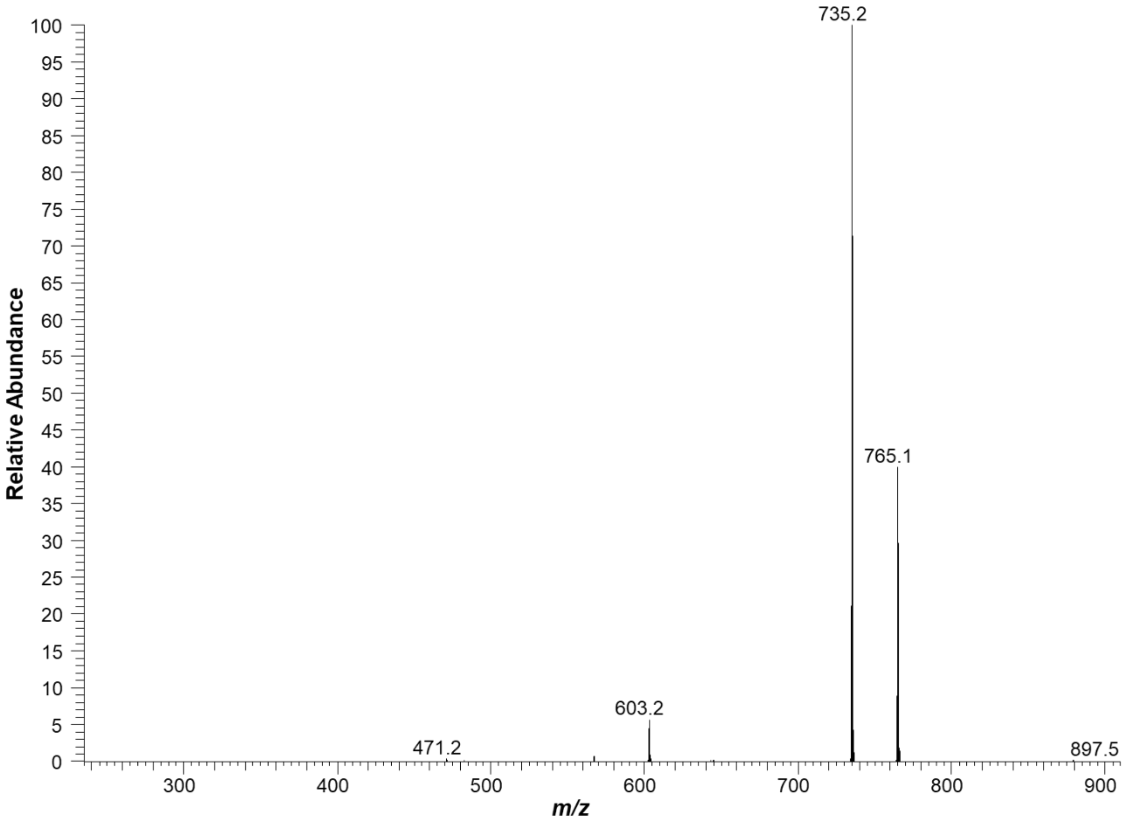

Figure S7: MS profile of extracts in the region of UV absorption compounds (0-5mi

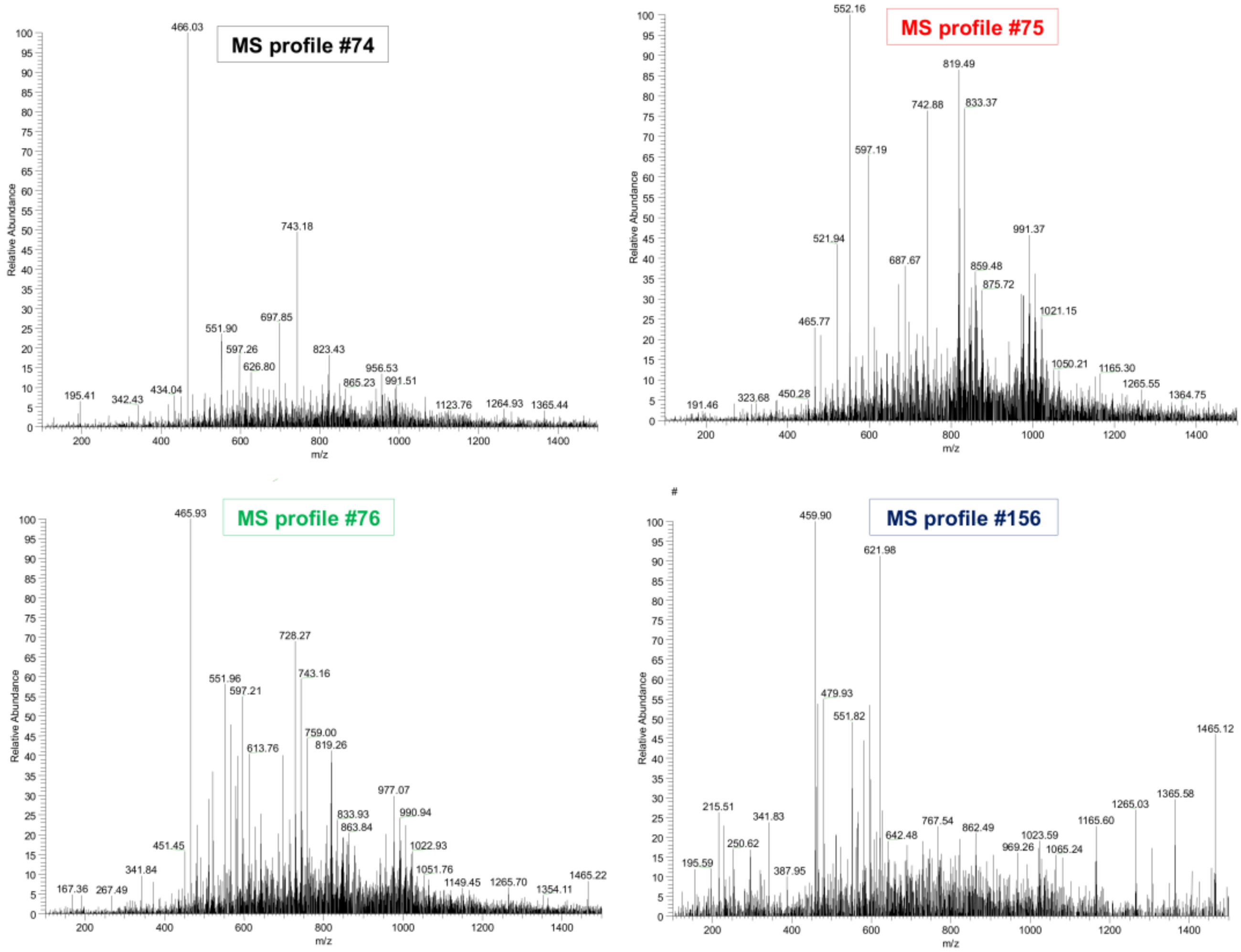

#74 - Aqueous bark extract prepared in laboratory scale; **#75** - Ethanol bark extract, prepared after the aqueous extraction of 74. **#76** - Ethanol bark extract, prepared by percolation; **#156** - Aqueous bark extract, prepared in a pilot-scale industrial plant.

Figure S8: MS/MS spectra of compound **7** at *m/z* 621.4 $[M-H]^-$

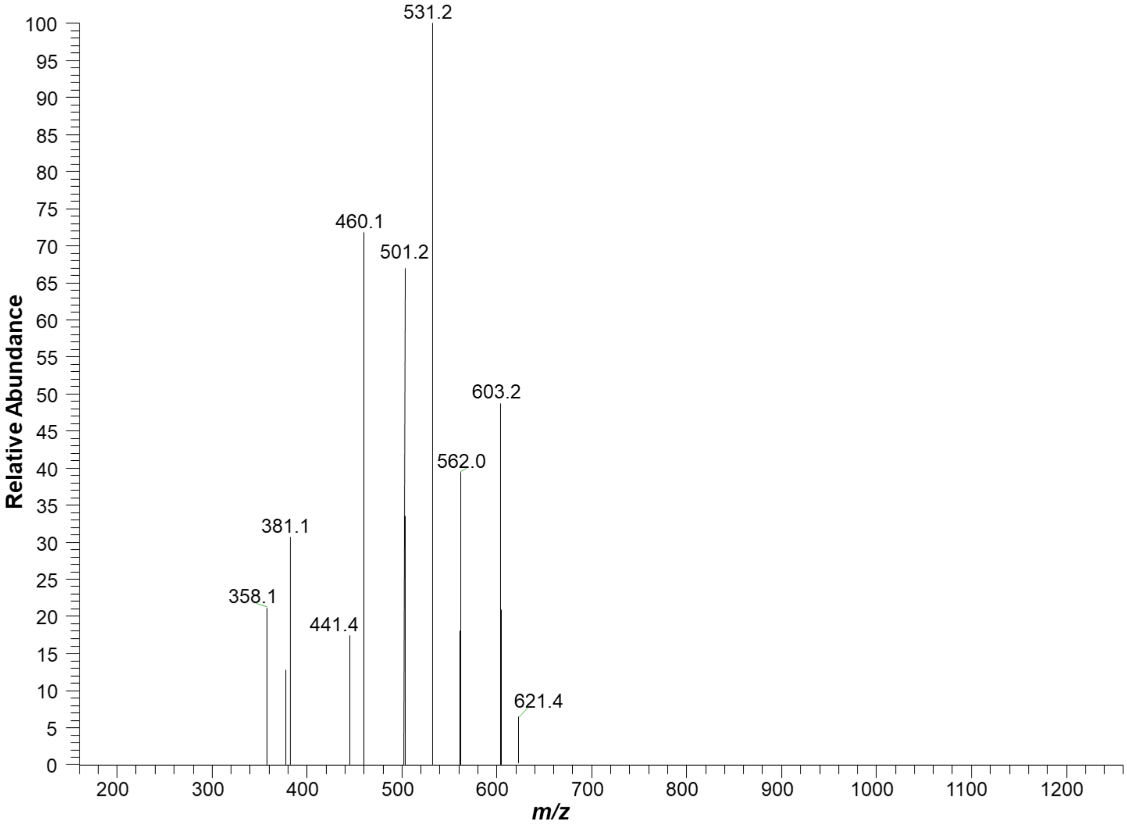

Figure S9: MS/MS spectra of compound **8** at *m/z* 597.5 $[M-H]^-$

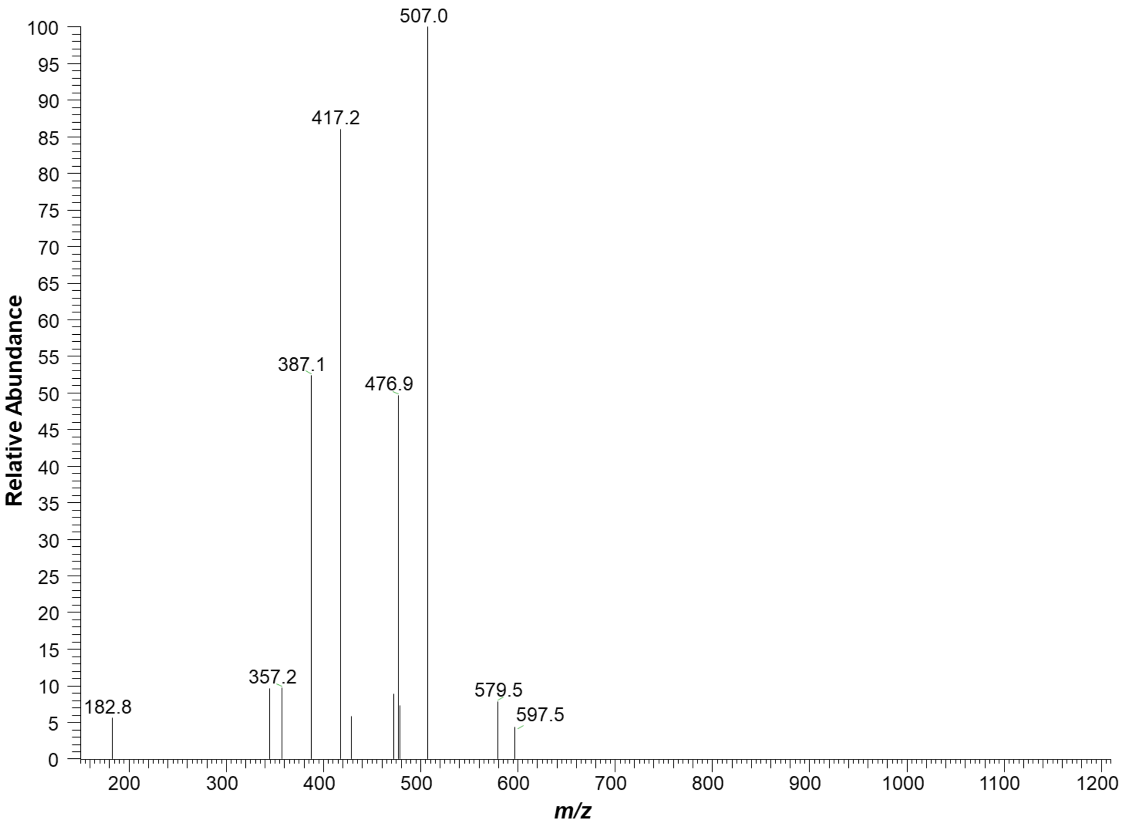

Figure S10: MS/MS spectra of compound **9** at *m/z* 465.7 $[M\text{-}H+H_2O]^-$ (water adduct)

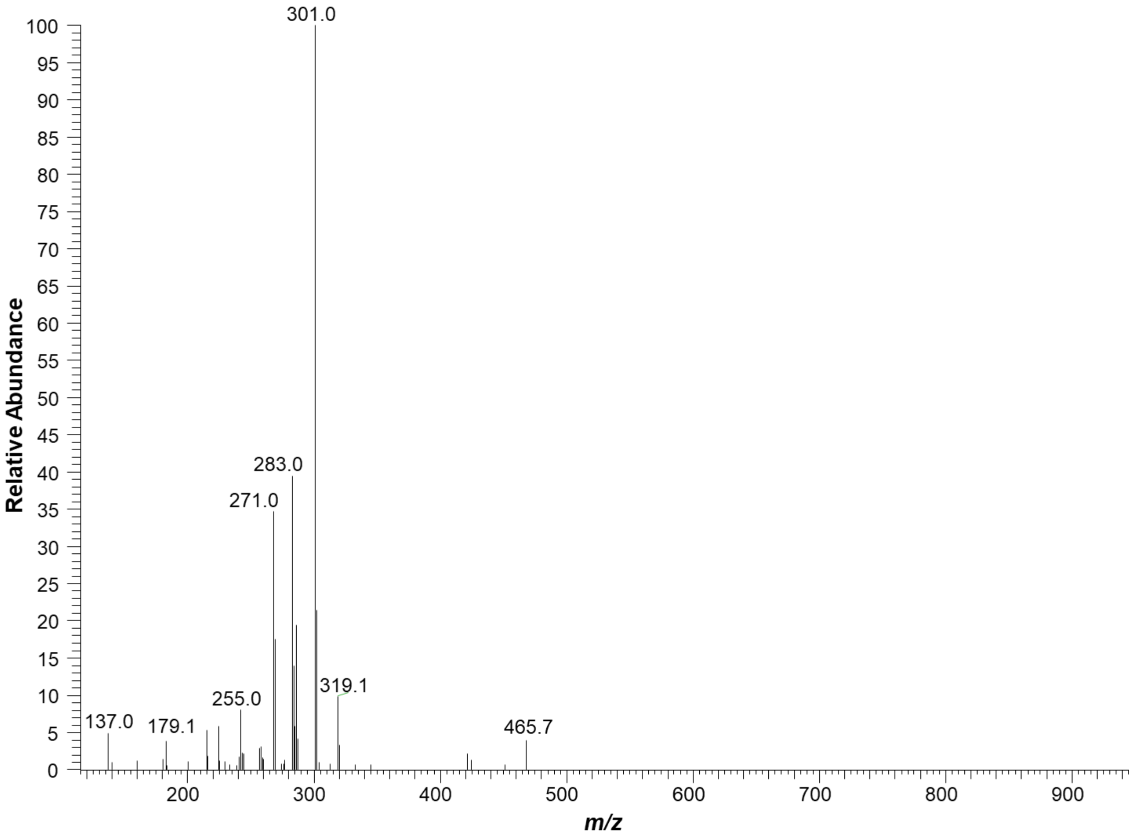

Supplement: Supplementary file 1 [file molecules-28-03159-s001.zip › molecules-2304098-supplementary.pdf]
